# Supplementary material for: Causal Relationship between Aging and Anorexia Nervosa: A White-Matter-Microstructure-Mediated Mendelian Randomization Analysis
Source: Biomedicines. 2024 Aug 16;12(8):1874. doi: 10.3390/biomedicines12081874 (PMC11351342; doi:10.3390/biomedicines12081874)
Supplement: Supplementary file 1 [file biomedicines-12-01874-s001.zip › Supplementary Figure/Figure S8/Leaveoneout plot of MR analysis for TL to ubm-b-1893.pdf]

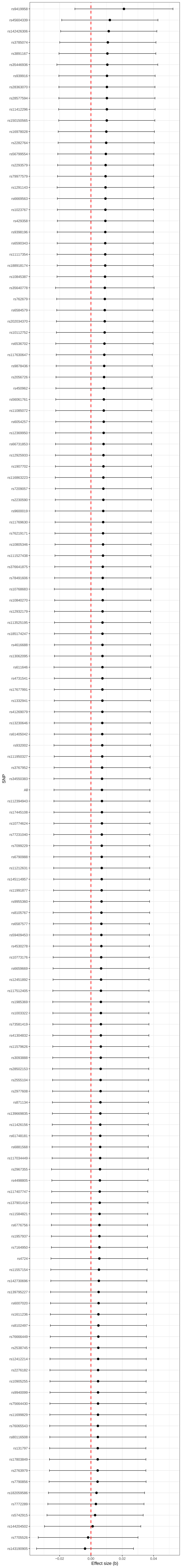
rs9419958
rs45604339
rs142426306
rs3785074
rs3891167
rs35446936
rs939916
rs28363070
rs28577594
rs11412296
rs150150565
rs16978028
rs2282764
rs56799554
rs2293579
rs79977579
rs1291143
rs6669563
rs1023767
rs429358
rs9398196
rs6590343
rs11117354
rs188918174
rs10845387
rs35640778
rs762679
rs6584579
rs202034370
rs10112752
rs6536702
rs117630647
rs9878436
rs2056726
rs450962
rs56061761
rs11085072
rs6054257
rs12369950
rs66731853
rs12925933
rs1907702
rs116863223
rs7209057
rs2230590
rs9600019
rs11769630
rs76219171
rs10805346
rs111527438
rs376641875
rs78491606
rs10768683
rs10840270
rs12932179
rs113525195
rs185174247
rs4616688
rs13062095
rs611646
rs4731541
rs17677991
rs1332941
rs41269079
rs13230646
rs61405042
rs932002
rs111950327
rs3767952
rs34550383
All
rs112394943
rs17445108
rs10774624
rs77231040
rs7099229
rs6790988
rs12212633
rs145114957
rs11991877
rs9955360
rs8105767
rs6587577
rs59409453
rs4530278
rs10773176
rs6659669
rs12451892
rs117512405
rs1985369
rs1003322
rs73581419
rs41304632
rs11579626
rs3093888
rs28502153
rs2555104
rs2977608
rs871134
rs139669835
rs11426156
rs61748181
rs6881568
rs117034449
rs2967355
rs4498805
rs117407747
rs137901416
rs11584821
rs6776756
rs1957937
rs7164950
rs4724
rs11557154
rs142730696
rs139795227
rs6007020
rs1611236
rs8102497
rs76666449
rs2538745
rs12412214
rs2276182
rs10905255
rs9940099
rs75664430
rs11699829
rs76065543
rs80116508
rs131797
rs17803849
rs2763979
rs7790856
rs182059586
rs7772289
rs5742915
rs144204502
rs7705526
rs143190905
SNP
-0.02
0.00
0.02
0.04
Effect size (b)
